# Evaluation of Antibacterial Synergism of Methanolic Extract of Dracocephalum kotschyi and Trachyspermum ammi

**DOI:** 10.21315/mjms2021.28.6.7

**Published:** 2021-12-22

**Authors:** Mohadeseh ZAREI YAZDELI, Ciamak GHAZAEI, Elaheh TASALLOT MARAGHI, Ashraf BAKHSHI, Marzieh SHUKOHIFAR

**Affiliations:** 1 Department of Medical Laboratory Sciences, Kashan Branch, Islamic Azad University, Kashan, Iran; 2 Department of Microbiology, University of Mohaghegh Ardabili, Ardabil, Iran; 3 Department of Microbiology, School of Biology, College of Science, University of Tehran, Tehran, Iran; 4 Shahid Beheshti Hospital, Kashan University of Medical Sciences, Kashan, Iran; 5 Diabetes Research Center, Shahid Sadoughi University of Medical Sciences, Yazd, Iran

**Keywords:** antibacterial activity, methanolic extracts, Lamiaceae, Trachyspermum ammi, food preservatives, synergistic effect

## Abstract

**Background:**

Chemical preservatives are now used in various foods to increase shelf life and maintain quality instead of its natural extracts with anti-bacterial properties from plants can be used*.* Hence this research was planned to evaluate and study the synergistic antibacterial effect of the methanolic extracts of *Dracocephalum kotschyi* (*D. kotschyi*) and *Trachyspermum ammi* (*T. ammi*) against standard pathogenic bacteria like: *Pseudomonas aeruginosa* (*P. aeruginosa*), *Shigella dysenteriae* (*S. dysenteriae*),* Escherichia coli* (*E. coli*) and* Staphylococcus aureus* (*S. aureus*).

**Methods:**

The methanolic extract of *D. kotschyi* and *T. ammi* was prepared by the Soxhlet method. The minimum inhibitory concentration (MIC) of this methanolic extracts were determined by the microdilution method. Thus, by determining the amount of fractional inhibitory concentration index (FICI), the interaction between the methanolic extracts of *D. kotschyi* and *T. ammi* on the pathogenic bacteria was determined.

**Results:**

In this study, the MIC of the extracts of *D. kotschyi* and *T. ammi* on the pathogen; *S. aureus* was equal to 6.25 mg/mL and 12.5 mg/mL for* S. dysenteriae*, *E. coli* and *P. aeruginosa*. Hence, the combination of methanolic extracts of these plants shows a synergistic antibacterial effect (FICI < 0.5), on all tested pathogenic microorganisms was proved.

**Conclusion:**

Due to the antimicrobial synergistic effect and cost-effective production process of methanolic extracts of *D. kotschyi* and *T. ammi*, they are used as natural preservatives and flavouring agents to preserve foods.

## Introduction

To control the growth of pathogenic bacteria in food, it is important to use preservatives and antimicrobial compounds for public health. Chemicals are used to preserve food from the growth of germs or killing and destroying harmful microorganisms due to side effects, the demand for natural plant preservatives without side effects, which would create an inappropriate taste and odour in food and the human immune system has increased (1). More than 10% of known plant species have medicinal uses. According to the World Health Organization (WHO), 80% of people living in developed countries more or less use medicinal plants for treatment. Medicinal plants with many secondary metabolites are the active ingredients of many drugs (2). Thus, these herbs with antibacterial, antifungal and antiparasitic activities are used to treat many diseases by stimulating the immune system, anti-inflammatory and antioxidant activity, increasing digestion and absorption of nutrients (3). It is necessary to study the active compounds of medicinal plants in each geographical area to discover the effective substances with antimicrobial properties. In this study, the antimicrobial effect of two medicinal plants: *Dracocephalum kotschyi* (*D. kotschyi*) and *Trachyspermum ammi* (*T. ammi*) was investigated.

*D. kotschyi* species is a perennial plant belonging to the family *Lamiaceae*. It is a semi-woody plant with a height of 10 cm–20 cm, petiolate leaves, ovate and large flowers, white or yellowish-white, integrated into cycles located in the stem bands as an end cluster. This plant contains many compounds such as tannins, terpenoids, saponins, sterols and flavonoids that can be used as an extract of this plant, to treat many diseases. The essence of this plant in terms of aroma and flavouring has a variety of applications in many industries such as cosmetics and perfumes, beverages, ice cream, confectionery, food products etc. (3–4). *T. ammi* belonging to the family *Apiaceae* is herbaceous, hairless and fragrant plant with a raised stem 20 cm–50 cm in height. The medicinal organ of this plant consists of its seeds, which are small, oval and yellow and are consumed dry and ripe.

Also, the most important chemical compounds of this plant include thymol, cymene, α-pinene, dipentene, γ-pinene and carvacrol. Seeds of* T. ammi* are used as a flavouring agent in chocolate, syrups, medicine and in food. In traditional medicines, *T. ammi* is being used orally as an analgesic, anti-asthmatic, expectorant, against nausea and also used in the treatment of rheumatic pain (5–6). Due to the presence of various phytochemicals with significant antibacterial properties in *D. kotschyi* and *T. ammi* and the rise of the global problem of drug resistance in bacteria on the other hand, there is a need to carry out laboratory studies, to determine the effects of such natural substances on the pathogenic microorganisms and to evaluate whether they have a potential to be used as alternatives to the chemicals, antibiotics or chemical drugs.

Previous research has shown the combined use of many herbal extracts which has more antimicrobial effect compared to the use of a single herbal extract. This study evaluated the synergistic antimicrobial effect of herbs like *D. kotschyi* and *T. ammi* (7).

## Methods

This experimental study was conducted with the code of ethics, from May 2019 to November 2019, in the microbiology laboratory of the Islamic Azad University, Kashan branch.

### Collect and Identify the D. kotschyi and T. ammi

*T. ammi* seeds were collected from the Sistan region. The genus and the species of *T. ammi* with herbarium number 1-0303-293 were approved by the expert of Medicinal Plants Research Center of Shahid Sadoughi University of Medical Sciences, Yazd, Iran. Also, the leaves and branches of the *D. kotschyi* from Fereydun Shahr were collected from the heights areas of Isfahan. The genus and the species of the *D. kotschyi* with herbarium code 309 PMP were identified at Shahid Beheshti University and then were approved by the Agricultural and Natural Resources Research Center of Isfahan Province. Both the plants were coded and completely identified by the systematic experts based on their specifications. The collected plants were placed in the open air to dry them completely. The dried plants were then pulverised with a mixer (4, 6).

### Preparation of Methanolic Extract

About 30 g of each plant (seeds of *T. ammi* and leaves and branches of *D. kotschyi*) were individually wrapped in a soft and clean filter paper and placed inside the Soxhlet machine. Extraction was performed with 100 mL of 96% methanol solvent. After 8 h, the extract was filtered with an antimicrobial filter of 0.45 μm and concentrated with a vacuum distillation device (rotary) and dried in the oven at 40 °C to separate the solvent from the extract. Finally, the pure extract obtained was stored in the refrigerator for further microbial tests, away from light (7).

### Preparation of Standard Strains

To evaluate the antibacterial properties of *D. kotschyi* and *T. ammi*, lyophilised vials of *Shigella dysenteriae* (*S. dysenteriae*) (American type culture collection [ATCC]: 1188), *Pseudomonas aeruginosa* (*P. aeruginosa*) (ATCC: 1310), *Staphylococcus aureus* (*S. aureus*) (ATCC: 1112) and *Escherichia coli* (*E. coli*) (ATCC: 25922) was provided from the Scientific and Industrial Research Center of Iran. Then, from a 24-h culture of bacteria grown on Müller-Hinton broth culture medium (HiMedia, India), a microbial suspension containing 1.5 × 10^8^ bacterial cells was prepared and in a spectrophotometer (UNIC-UV-2100, USA) at 630 nm in the range of 0.08–0.1 was approved. Then 0.2 mL of this suspension was added to 19.8 mL of Müller-Hinton broth culture medium to achieve a concentration of 1.5 × 10^6^ bacterial cells (8–9).

### Preparation of Different Extract Concentrations of D. kotschyi and T. ammi

To prepare different concentrations of methanolic extracts, 0.2 g of each dry extract was weighed separately and added to a sterile test tube containing 4 mL of Müller-Hinton broth culture medium containing 20% dimethyl sulfoxide (DMSO). Thus, the concentration of the extract was achieved 50,000 μg/mL. A sterile extract was assessed for the absence of turbidity in the broth after the incubation period. Seven different dilutions: i) 25,000 μg/mL (50%); ii) 12,500 μg/mL (25%); iii) 6,250 μg/mL (12.5%); iv) 3,125 μg/mL (6.25%); v) 1,562.5 μg/mL (3.125%); vi) 781.25 μg/mL (1.56%) and vii) 390.62 μg/mL (0.78%) were prepared from the original extract of 50,000 μg/mL (100%) and all these eight different concentrations were assessed for their antibacterial properties (10, 11, 12).

### Investigation of Antibacterial Activity of Extracts by Diffusion Method in Wells

In the well-diffusion method, 100 μL of microbial suspension with a concentration of 1.5 × 10^6^ colony forming unit (CFU)/mL was spread evenly on the surface of the Müller-Hinton agar medium. Then, wells with a diameter of 6 mm and a distance of 2.5 cm were created on the plate surface by a sterile pasteuriser pipette and filled with 100 μL of different concentrations of the extract. The negative control was the DMSO solution and the imipenem antibiotic was used as a positive control. All inoculated media were exposed to 37 °C for 24 h and examined for the formation or non-formation of the growth inhibition zone around the wells and the diameter of the growth inhibition zone is measured in mm which was repeated three times for each extract and finally the average diameter of the growth inhibition zone was calculated (1, 13).

### Measurement of Minimum Inhibitory Concentration and Minimum Lethal Concentration of D. kotschyi and T. ammi by Microdilution Method

After preparing different concentrations of the extract, minimum inhibitory concentration (MIC) and minimum lethal concentration (MLC) values were determined by the microtitre plate. For this purpose, 100 μL of each concentration 1.5 x 10^6^ CFU/mL was added to the wells of the first to tenth columns of 96 sterile house micro-plates (each column corresponds to one concentration, respectively). To column 11 was added as a negative control 200 μL of the Müller Hinton broth medium. One hundred microlitre of microbial suspension and 100 μL of the Müller-Hinton broth were added to column 12 as a positive control. After inoculation, the light absorption of the wells was read by the ELISA reader at a wavelength of 450 nm. The micro-plate was incubated at 37 °C. After 24 h the light reabsorption of the wells was read by the ELISA reader at a wavelength of 450 nm (14). Each experiment was performed three times and the average of the results was calculated.

MIC was recorded for the lowest concentration of the plant’s extract (alone) or extracts (combination), which showed visible inhibition of the growth of the testing pathogenic bacteria. To determine the amount of MLC, 20 μL of the three clear pre-MIC cells were cultured on Müller-Hinton agar medium. After 24 h, the lowest concentration of the extract in which the bacteria did not grow was reported as the MLC (13, 14).

### Evaluation of the Combined Effect of Methanolic Extract of D. kotschyi and T. ammi in Preventing the Growth of Standard Pathogens

Fractional inhibitory concentration index (FICI) was determined to investigate the antimicrobial interaction of methanolic extract of *D. kotschyi* and *T. ammi*. A conventional checkerboard method was used to determine the FICI and the FICI was calculated from the following equation:


$$\sum \text{FICI}={\text{FICI}}_{\text{A}+}\;{\text{FICI}}_{\text{B}}={\text{MIC}}_{\text{AB}}/{\text{MIC}}_{\text{A}}+{\text{MIC}}_{\text{BA}}/{\text{MIC}}_{\text{B}}$$

where

MIC_AB_: minimum inhibitory concentration of *T. ammi* along with* D. kotschyi*
MIC_A_: minimum inhibitory concentration of *T. ammi*
MIC_BA_: minimum inhibitory concentration of *T. ammi* along with* D. kotschyi*
MIC_B_: minimum inhibitory concentration of *D. kotschyi*

In this formula, the sum of the FICIs of the substances A and B represents the following:

∑FICI values indicate that, FICI ≤ 0.5 = synergistic effect, FICI > 0.5 but ≤ 1 = additive effect, FICI > 1 but ≤ 4 = indifferent effect, and FICI > 4 = antagonistic effect (15, 16, 17).

First, two consecutive concentrations of methanolic extract of *D. kotschyi* and *T. ammi* were prepared separately as in the previous section.

In each row, 50 μL of 100,000 μg/mL–781.25 μg/mL *T. ammi* extract was poured from left to right, respectively.

In each column, 50 μL from top to bottom, concentrations of 100,000 μg/mL– 781.25 μg/mL, *D. kotschyi* methanolic extract was poured. Finally, we had a microplate in which each of the concentrations of one extract was in combination with other extract. Then, 100 μL of 10^6^ CFU/mL dilution of bacterial suspension was added to each well. Thus, the final volume of each well reached 200 μL. Four wells were also considered as positive controls (100 μL Müller-Hinton agar medium and 100 μL microbial suspension), each well was mixed and the microplate was incubated at 37 °C for 24 h. About 50 μL of triphenyl tetrazolium chloride (5 mg/mL) was poured into all microplate wells and re-incubated for 3 h at 37 °C, a concentration higher than the last concentration that gave the tetrazolium red colour, which was considered as a combination bacterial MIC (11, 17, 18).

### Isolation and Identification of the Constituents of the Extract

To isolate and identify the constituents of *T. ammi* and *D. kotschyi* extracts, a volume of 1 μL of each extract was injected into the mass spectometry/gas chromatography apparatus. Hewlett Packard 6890 model, injection site temperature 240 °C, thermal programming: 50 °C–300 °C, column type: HP-5MS, carrier gas: helium, gas flow rate: 1.5 mL/min, column length: 30 m, internal diameter: 250 μm and ionisation energy: 70 EV. According to the exit pattern of normal alkanes, inhibition index and Quats index, and their adaptation to library patterns, the spectrum is interpreted for each object and major constituents of the extracts were identified (4–5).

## Statistical Analysis

All tests were performed with three replications and the differences between the data were analysed using the one-way analysis of variance (ANOVA) and SPSS version 18 for Windows at a significance level of 0.05 and Bonferroni paired test with SPSS version 18. A significance test level (*P* < 0.05) was used to interpret the data. In this test, inhibitory zones obtained for the different concentrations of *D. kotschyi* and *T. ammi* (in mm) on different food spoilage microorganisms, were compared with the results obtained for the positive control (imipenem) and represented as a *P*-value at a significance level of 0.05 (*P* < 0.05).

## Results

The results of the present study show that the methanolic extracts of *D. kotschyi* and *T. ammi* have an inhibitory effect on all studied bacteria.

Table 1 shows the average diameter of growth inhibition halo of methanolic extract of *D. kotschyi* and *T. ammi* on different bacterial isolates (in mm). As the ANOVA test shows, the size of the diameter of the growth inhibition zone with different concentrations of the extract has a direct effect (*P* < 0.05). The concentration of methanolic extracts of *D. kotschyi* and *T. ammi* increases its antimicrobial activity and antibacterial effect. This process of effect on this strain indicates that methanolic extracts in concentrations of 50,000 μg/mL–6,250 μg/mL have an antibacterial effect, on standard strains and at concentrations less than 6,250 μg/mL no inhibition growth halo were observed for both the plants. To compare the diameter of the growth inhibition zone at concentrations of 50,000 μg/mL–6,250 μg/mL in the methanolic extracts of *D. kotschyi* and *T. ammi*, ANOVA was used where significant differences were observed.

Based on the results of the Bonferroni test, the diameter of the growth inhibition zone in imipenem was significantly larger than the concentrations of 50,000 μg/mL–6,250 μg/mL of the extracts (*P* < 0.05). According to Bonferroni test, the diameter of the growth inhibition zone was significantly different between the concentrations of 12,500 μg/mL and 25,000 μg/mL; 12,500 μg/mL and 50,000 μg/mL; 25,000 μg/mL and 50,000 μg/mL; as well as the concentrations of 6,250 μg/mL and 25,000 μg/mL; 6,250 μg/mL and 12,500 μg/mL; and 6,250 μg/mL and 50,000 μg/mL (*P* < 0.05). Between the four concentrations of the extracts, the diameter of the growth inhibition zone at a concentration of 50,000 μg/mL was significantly larger than other concentrations (*P* < 0.05). Hence statistical analysis results were found to be almost similar for the both the extracts, used in the form of single dose of the methanolic extracts obtained from *D. kotschyi* and *T. ammi*.

Table 2 shows that the MIC of methanolic extract of *D. kotschyi* and *T. ammi* individually for Gram-negative bacteria was 12,500 μg/mL (12.5 mg/mL) and the MIC was 25,000 μg/mL (25 mg/mL) for *S. dysenteriae*, *E. coli* and *P. aeruginosa*. Both extracts had the greatest effect on Gram-positive bacteria (*S. aureus*) with MIC value of 6,250 μg/mL (6.25 mg/mL). Also, the MIC of methanolic in the combined state was lower 1562.5 μg/mL (1.5625 mg/mL) for *S. aureus* and 3,125 μg/mL (3.125 mg/mL) for *S. dysenteriae*, *E. coli* and *P. aeruginosa* bacteria than that of the single state.[Table t3-07mjms2806_oa]

In general, the results of this section show that according to the FICI, the combination of methanolic extracts of *D. kotschyi* and *T. ammi* has a synergistic effect against bacteria (∑FICI < 0.5).

### Chemical Composition of Methanolic Extract of T. ammi and D. kotschyi

The main chemical constituents of *T. ammi* extract were thymol 48.96%, p-cymene 23.73% and gamma-terpinene 15.98%. The main chemical constituents of *D. kotschyi* extract were linalool 12.08%, α-pinene10.34%, geranyl acetate 10.27%, geranial 9.55%, nerol 8.90% and limonene 6.95%.

## Discussion

Numerous reports of contamination from contaminated food reveal the need for constructive solutions for food safety and health. Although the inhibitory effects of the spices, plant extracts and essence have long been known; recently, special attention has been paid to the effects of the essence and aromatic plant extracts on pathogens and microorganisms which are responsible for the food spoilage. *D. kotschyi* and *T. ammi* are among the plants that are widely used in traditional medicine (19–20). In this study, both qualitative (well propagation) and quantitative (micro-dilution) methods showed that *D. kotschyi* and *T. ammi* extract inhibits the growth of the tested bacteria. The result of the study shows that in the well-method, the amount of extracts’ concentration has a direct effect on the size of the growth inhibition zone (*P* < 0.05) and also growth inhibition is different in different bacteria thus, methanolic extracts have a differential impact on the different types of bacteria.

The differences observed in bacteria and antimicrobials is due to the structures of the microorganisms and also less effective in Gram-negative bacteria due to the structural differences between the cell walls of these two groups of bacteria. Gram-negative bacteria have an outer membrane that acts as a barrier to the passage of large hydrophobic molecules (21). In addition, most of the active compounds in extracts are hydrophobic, and these substances are not able to enter and access the active sites inside Gram-negative bacteria, and they have enzymes in the periplasmic space that can decompose foreign molecules. These factors might have affected the antibacterial activity of the herbal extracts and hence found to be less effective against Gram-negative bacteria than that of Gram-positive bacteria (19, 20, 22, 23).

Moridi Farimani et al. (22) studied antimicrobial effect with the help of antibacterial assay of essential oils obtained from *D. kotschyi* and carried out broth micro-dilution method to determine the MIC of the samples against Gram-positive and Gram-negative bacteria, *S. aureus* (ATCC: 25923) and *E. coli* (ATCC: 25922), respectively. Their study too showed that *S. aureus* is the most sensitive microorganism to the essential oil obtained from *D. kotschyi* and this result showed similarity with Moridi Farimani et al.’s (22). The most important compounds in the *D. kotschyi* include thymol, carvacrol, menthol, beta-pinene and linalool. These compounds have antibacterial activity, the antibacterial effect of *D. kotschyi* extract in the present study are similar (4, 19, 22).

In Ashrafi’s (24) study, the essence of *D. kotschyi* on *P. aeruginosa* (ATCC: 27853), *S. aureus* (ATCC: 12600), *E. coli* (ATCC: 11775), *Klebsiella pneumoniae* (*K. pneumoniae*) (ATCC: 27853) has created a growth inhibition zone of 10 mm, 21 mm, 15 mm and 18 mm, respectively. MIC of the essence for *P. aeruginosa* (ATCC: 27853), *S. aureus* (ATCC: 12600), *E. coli* (ATCC: 11775) and *K. pneumoniae* (ATCC: 700603) was 320 μg/mL, 160 μg/mL, 320 μg/mL and 640 μg/mL, respectively. The antibacterial properties of Ashrafi’s study showed lesser MIC values as compared to the present study. The reason for the difference in the results is due to differences in the bacterial strain. Because the composition of essence obtained from a particular plant species varies based on the geography of the region (24, 26).

In 2018, Khan and Jameel (6) examined of all the extracts, the methanolic and ethanolic extracts of *T. ammi* seeds had the highest antibacterial properties than that of aqueous extract (6). Essential oil and different types of extracts of *T. ammi*, have shown their antibacterial effects against some food spoilage microorganisms and also against antibiotic-resistant microorganisms (23, 27, 28). In 2019, Jafari-Sales et al. (29) investigated the antibacterial effects of methanolic extract of *Carum copticum* (*C. copticum*)* L*. on *S. aureus* and *P. aeruginosa*. The results showed that the antibacterial effects of *C. copticum L.* methanolic extract against Gram-positive bacteria were higher than Gram-negative bacteria so that, the largest diameter of growth inhibition zone was observed in *S. aureus* (20 mm). The results of our study are consistent with the results of Jafari-Sales et al. (29) and, Khan and Jameel (6).

As mentioned, the most important active ingredient in *T. ammi* is phenolic compounds such as thymol and other compounds such as β-para-cement and alpha-terpinene. Phenolic compounds have various properties, including their antibacterial properties. The mechanism of phenolic compounds acts on bacteria through cell membrane lysis, as well as binding to bacterial cell compounds and inactivating enzymes, binding to adhesins, and forming cell wall complexes (29).

In 2018, Heidari Soureshjani et al. (30) evaluated the effect of *T. ammi* essence on *E. coli* bacterium. The results showed that the MIC of *T. ammi* essence against *E. coli* was 1562.5 μg/mL. Also, in the 2019 study by Javan et al. (20), the MIC of *T. ammi* essence on *S. aureus* was 500 μg/mL. Comparing the results, we find that the essence of *T. ammi* in Heidari Soureshjani et al.’s (30) and Javan et al.’s (20) studies have a better antibacterial effect than our study. This difference may be due to differences in the types of extracts used in those studies. In a study by Sharifi Mood et al. (23), the MIC essence of *T. ammi* on *S. aureus* was reported to be 1.205 mg/mL. In this study, the important components of the essence of *T. ammi* were thymol, para-cement, gamma-terpinene, carvacrol and beta-pinene (23).

In a 2016 study by Hosseinkhani et al. (31), the MIC of *T. ammi* essence for various foodborne pathogens ranged from 12.5 μg/mL to 5.4 μg/mL. Thus, results showed that *T. ammi* essence was more sensitive to Gram-positive bacteria than Gram-negative bacteria, which is consistent with the results of this study. Oroojalian et al. (32) also investigated the antibacterial effect of *T. ammi* essence against some important food pathogens by micro-dilution method and stated that its MIC is in the range of 0.5 mg/mL–0.03 mg/mL which is consistent with the results of this study (32).

In another study, Bashyal and Guha (33) found different phytochemical constituents in the different extracts like methanol, acetone, chloroform and aqueous extracts of *T. ammi* seeds and tested their antimicrobial activity against *E. coli* with 70 μL of each extract. They found that methanolic extracts of *T. ammi* has highest antibacterial activity against *E. coli* than that of other three extracts. Phytochemical analysis study showed that methanolic extract contains alkaloids, carbohydrates, saponins, flavonoids, proteins and glycosides. And in aqueous extract only alkaloids were absent. In acetone extract, carbohydrates, saponins, flavonoids, terpenoids and glycosides were present, while in acetone extract only saponins and flavonoids were present. Hence, they concluded that these different phytochemicals might be contributing in the antibacterial properties of *T. ammi* seeds (33).

Different studies like Oroojalian et al.’s (32), Sharifi Mood et al.’s (23) and Hosseinkhani et al.’s (31), found that the important components of *T. ammi* essence included thymol, para-cementone, gamma-terpinene, carvacrol and beta-pinene. These studies have investigated that thymol is involved in destruction of the bacterial membrane integrity, which causes leakage of intracellular material leading bacterial cell’s death.

Low solvent toxicity, ease of evaporation at low temperature and no formation of bonds with active compounds in the extract or their degradation are suitable features of the solvent in the preparation of plant extracts. Therefore, depending on the type of target, which is polar or non-polar extraction material, the type of solvent will also be different. Almost all known antimicrobial compounds are aromatic or organic compounds and are mostly extracted through ethanolic and methanolic solvents. Also, extracts prepared with organic solvents have a more stable and more antimicrobial effect (6, 19). In Heidari Soureshjani et al.’s (30) study, the calculation of differential inhibitory concentration (FIC index) indicated that there was no interaction between the essence of *T. ammi* and fennel against *E. coli,* may be due to different herbs used.

The results of the Javan et al.’s (20) study showed that the combined effect of *T. ammi* essence and ethanolic extract of propolis has a synergistic effect in inhibiting the growth of foodborne pathogenic bacteria. Sadiki et al. (16) evaluated the combined effect of *Myrtus communis* and *Thymus vulgaris* essential essence against *E. coli* and *S. aureus*. The FIC index showed the synergistic effect of both essential oils against both bacteria (16). In 2016, Bakarnga-Via et al. (34) investigated the effect of combined extracts of various parts of *Annona senegalensis* on antibacterial and antifungal activities. The combination of the hydro-ethanolic extract of the bark (BHEtOH) with the ethanolic extract of the leaves (LEtOH) showed additivity against *S. aureus* (FICI = 1) and synergistic interaction against *E. coli* (FICI = 0.25), while FICI of the combinations of LEtOH/TwH_2_0_2_, StH20/TwH_2_0_2_, LEtOH/BH_2_0_2_, and StH_2_0_2_/BH_2_0_2_ against four yeast strains showed antagonistic interactions (FICI > 4) (34).

In 2020, Effah-Yeboah et al. (35) studied the antimicrobial activity of the combined effect of *Kalanchoe crenata* and *Vernonia amygdalina* on *Salmonella sp*. Calculation of differential inhibitor concentration (FIC index) indicated that there was no interaction between the essences of the two plants against the tested bacteria. When combinations of several antimicrobials act simultaneously on a microbial population, they may result in an increased (synergistic), decreased (antagonistic) or indifferent antimicrobial response compared to their individual effects. One of the hypotheses describing the synergistic state is the existence of one compound to accelerate and facilitate the binding of the other compound to the site by making changes in the cell structure. Compared to the results of this study and Javan et al.’s (20) study where the synergistic effect of the two different herbal extracts can be seen, in the other study of Bakarnga-Via et al. (34), different types of effects such as additive, synergistic, antagonistic can be seen for the different types extracts obtained from different parts of the same plant.

Antioxidant effects of the *T. ammi* that can help in the preservation of the food by inhibiting the lipid-auto-oxidation process in foodstuffs and preventing the production of rancid and stale flavours in the food (28). Also, *D. kotschyi* has flavonoids such as luteolin, apigenin, cirsimaritin, xanthomicrol and rosmarinic acid and many of them can be explored for their other useful characteristics like anti-inflammatory, anti-oxidant and anti-cancer activities (36, 37, 38).

In the present study, the combined effect of *D. kotschyi* and *T. ammi* methanolic extract was investigated. The results showed a synergistic action between methanolic extract of *D. kotschyi* and *T. ammi* on *S. dysenteria*, *P. aeruginosa*, *E. coli* and *S. aureus.* Results showed the combination of *D. kotschyi* and *T. ammi* methanolic extract has more antibacterial activity than the single state. The combined effect of the extract of *D. kotschyi* and *T. ammi* in hybrid technology of food storage, reduces the concentration of preservatives and by increasing the antimicrobial effects of the material maintains better product quality and health. Studies on the interaction of various compounds, especially essences and plant extracts in inhibiting the growth of microorganisms are limited. So far, no research has been done on the combined effect of the methanolic extracts of these plants.

## Conclusion

According to the results of well-diffusion and micro-dilution experiments and from the comparative analysis of these results, it can be claimed that a combination of the methanolic extracts of plants has a very favourable inhibitory effect on the growth of standard pathogenic strains of bacteria, especially against the *S. aureus*, which is effective in causing food poisoning by providing synergistic interaction. Synergistic effect might be due to the positive interactions between the components of the two herbal extracts that are used in the study as a combination, which result in the increased antibacterial activity and antimicrobial effect. The in vitro studies can help to determine the effective concentration of these extracts on the target bacteria and the clinical strains. Such studies might help to understand if there are any associated side effects with its use and also the precise formulation of the extracts, should be evaluated to plan further in vivo studies, accordingly. The favourable synergistic antibacterial activity obtained for the combination of the *D. kotschyi* and *T. ammi* herbal extracts against many pathogenic Gram-negative and Gram-positive bacteria pave a way for further studies, to evaluate their potential to be used as natural preservatives, to assure the safety of the food and to avoid usage of chemical preservatives.

## Figures and Tables

**Table 1:** Mean diameter of growth inhibition zone due to antimicrobial effect of different concentrations of methanolic extracts of *D. kotschyi* and *T. ammi* (mm)

Concentrations (μg/mL) sample	50,000	25,000	12,500	6,250	Positive control (imipenem)	*P*-value
Methanolic extract of *D. kotschyi* (mm)						
*S. dysenteriae* (ATCC: 1188)	17.75± 0.50	13.25 ±0.50	10.25± 1.50	9.80± 0.76	27.00± 1.81	0.022
*P. aeruginosa* (ATCC: 1310)	19.50± 0.50	17.50± 0.50	13.00 ±1.00	10.80 ±0.76	26.00 ±2.12	0.028
*E. coli* (ATCC: 25922)	18.50 ±0.50	16.50± 0.50	13.50± 0.50	9.50± 0.50	27.00± 2.12	0.018
*S. aureus* (ATCC: 1112)	20.75 ±0.95	19.50± 1.00	17.25± 1.50	11.5 ±1.00	28.00± 2.12	< 0.001
Methanolic extract of *T. ammi* (mm)						
*S. dysenteriae* (ATCC: 1188)	18.75 ± 0.50	16.25 ± 0.50	13.25 ± 1.50	9.80 ± 0.76	27.00 ± 1.81	0.022
*P. aeruginosa* (ATCC: 1310)	20.75 ± 0.50	18.50 ± 0.50	15.00 ± 1.00	9.80 ± 0.76	26.00 ± 2.12	0.034
*E. coli* (ATCC: 25922)	18.50 ± 0.50	16.50 ± 0.50	13.50 ± 0.50	9.50 ± 0.50	27.00 ± 2.12	0.041
*S. aureus* (ATCC: 1112)	22.75 ± 0.95	20.50 ± 1.00	16.25 ± 1.50	12.5 ± 1.00	28.00 ± 2.12	< 0.001

**Table 2:** MIC of methanolic extract of *D. kotschyi* and *T. ammi* alone and in combination on different bacteria (μg/mL)

Bacteria tested	*D. kotschyi* MIC (μg/mL) [alone]	*T. ammi* MIC (μg/mL) [alone]	Combination of *T. ammi* and *D. kotschyi* extract [for synergistic effect]
*S. dysenteriae* (ATCC: 1188*)*	12,500	12,500	3,125
*P. aeruginosa* (ATCC: 1310)	12,500	12,500	3,125
*E. coli* (ATCC: 25922)	12,500	12,500	3,125
*S. aureus (*ATCC: 1112*)*	6,250	6,250	1,562.5

**Table 3 t3-07mjms2806_oa:** An independent and combinational effect of methanolic extracts of *D. kotschyi* and *T. ammi* on standard pathogenic strains, represented as FICI

Bacteria tested	FICI of *T. ammi*	FICI of *D. kotschyi*	FICI of combinational extracts	Reaction
*S. dysenteriae* (ATCC: 1188)	0.25	0.25	0.5	Synergism
*P. aeruginosa* (ATCC: 1310)	0.25	0.25	0.5	Synergism
*E. coli* (ATCC: 25922)	0.25	0.25	0.5	Synergism
*S. aureus* (ATCC: 1112)	0.25	0.25	0.5	Synergism
